# The legacy of privilege: Social inheritance reverses sex differences in reproductive inequality in the spotted hyena

**DOI:** 10.1126/sciadv.aee7880

**Published:** 2026-07-23

**Authors:** Marta Mosna, Alexandre Courtiol, Philemon Naman, Oliver P. Höner, Eve Davidian

**Affiliations:** ^1^ Department of Evolutionary Ecology, Leibniz Institute for Zoo and Wildlife Research, Berlin, Germany.; ^2^ Department of Biology, Chemistry, and Pharmacy, Freie Universität Berlin, Berlin, Germany.; ^3^ Ngorongoro Hyena Project, Ngorongoro Conservation Area, Tanzania.; ^4^ Department of Evolutionary Genetics, Leibniz Institute for Zoo and Wildlife Research, Berlin, Germany.; ^5^ Institute of Evolutionary Science of Montpellier (ISEM), Université de Montpellier, CNRS, IRD, EPHE, Montpellier, France.

## Abstract

Inequalities in reproductive success among females and males shape natural and sexual selection. For sexually reproducing species, the Darwin-Bateman paradigm predicts greater reproductive inequality in males than females. A mechanism driving reproductive inequality in humans and other animals is social inheritance of privilege. Here, we show that this mechanism can reverse the male bias in reproductive inequality. Using a 29-year dataset spanning eight generations of spotted hyenas (*Crocuta crocuta*), a species in which social rank and privileges are inherited from the mother, we show that reproductive inequality was lower among females than males when estimated annually but increased more strongly in females over generations, quickly reversing the sex bias to females. Our findings suggest that social inheritance of privilege (i) is a powerful driver of sex differences in reproductive inequality and (ii) may explain why females are the more competitive sex in some species. We outline how reproductive inequality influences female and male reproductive strategies, intrasexual competition, and genetic evolution.

## Teaser

The social transmission of privilege from mother to daughters reverses typical male-biased reproductive inequality.

## INTRODUCTION

Females and males can differ profoundly in morphology, behavior, and life-history traits, and explaining this variation has been a central theme in evolutionary biology since Charles Darwin. The Darwin-Bateman paradigm provides the dominant framework to explain differences between the sexes (*1*–*4*). It proposes that anisogamy leads to greater reproductive inequality, i.e., the uneven distribution of reproductive success among individuals, in males than in females. When reproductive inequality is greater in males, males can gain more from additional matings, which may explain why, in many species, males are the more competitive sex and evolved pronounced weaponry and ornamentation (*5*). Reproductive inequality is therefore a key variable for testing the paradigm’s predictions and understanding the drivers and intensity of intrasexual competition (*5*, *6*).

Empirical studies confirmed that males show greater reproductive inequality than females in most species (*5*, *7*). However, female-biased reproductive inequality is not rare, and two pathways leading to it have been identified. First, in so-called sex-role reversed species, males can provide more parental care than females, and this can lead to intense female-female competition for access to mates (*8*), as in seahorses and pipefishes (*9*) and several bird species (*8*, *10*). Second, a few dominant females can monopolize reproduction, generating female-biased reproductive inequality and intense female-female competition, as in cooperatively breeding meerkats [*Suricata suricatta*; (*11*)] and some species of African starlings (Sturnidae) (*12*). Here, we propose a third pathway leading to female-biased reproductive inequality: the social inheritance of privilege.

Privilege—the differential access to inherited resources—is widespread in human and nonhuman societies and has long been recognized as a powerful driver of competition (*13*). It is also a powerful driver of reproductive inequality, although this is often overlooked (*13*–*15*). Privilege can arise from traits and resources that are inherited socially, such as the network of allies and social status [“relational wealth”; (*14*, *16*–*20*)] or materials [“material wealth”; (*21*–*23*)]. Like genetically inherited traits such as body size, strength, or weaponry (*24*–*26*), socially inherited privilege can translate into higher reproductive success and thus drive reproductive inequality. Privilege can persist across generations and be transmitted through the maternal lineage or the paternal lineage (*16*, *27*–*30*). Such sex-specific social inheritance can therefore modify reproductive inequality differently in males and females across generations and thus influence how natural selection shapes traits. For species in which privilege is inherited predominantly through females, social inheritance of privilege could reverse the typical sex bias predicted by the Darwin-Bateman paradigm.

Most studies quantifying reproductive inequality have done so over short timescales, such as a breeding season or a year, and less commonly over a lifetime (*7*, *31*). Using short timescales does not capture the potential long-term impact of social inheritance of privilege (*14*) and may explain why the inheritance of privilege has not been discussed as a potential driver of sex differences in reproductive inequality.

Here, we use a multigenerational dataset of spotted hyenas (*Crocuta crocuta*) to test whether social inheritance of privilege shapes reproductive inequality and potentially reverses the typical sex differences predicted by the Darwin-Bateman paradigm. Our 29-year dataset spans eight generations and contains 2743 spotted hyenas with a known mother from the free-ranging population of the Ngorongoro Crater in Tanzania. Spotted hyenas are an ideal system for this study because they live in clans organized by linear dominance hierarchies, offspring socially inherit their rank from their mother, and a high rank confers immense privileges (*32*, *33*). High-ranking individuals have priority access to all key resources, such as food, preferred dens, and den holes, and they benefit from greater social support than low-ranking individuals (*32*, *34*–*36*). High-ranking females enjoy these privileges throughout their lives, resulting in earlier reproduction, shorter interbirth intervals, higher cub survival, and longer lifespan compared to lower-ranking females (*37*, *38*). Sons of high-ranking females also enjoy fitness benefits (*39*), but these are more limited because, unlike daughters, sons typically disperse to another clan to reproduce (*40*) [but see (*41*)]. Thus, in contrast to females, males usually do not retain the privileges of their natal rank, and they do not transmit their rank or privilege to their offspring.

We expect reproductive inequality to be greater in males than in females when estimated over short timescales, consistent with the Darwin-Bateman paradigm. Due to the maternal inheritance of privilege, we further predict that inequality in the number of descendants will rise faster among females and eventually surpass inequality among males after several generations.

## RESULTS

### Reproductive success in female and male spotted hyenas

We calculated four measures of reproductive success for 287 females and 205 males that were monitored throughout their reproductive tenure: the (i) annual number of offspring, (ii) total number of offspring, as well as the number of (iii) grandoffspring and (iv) great-grandoffspring. The mean annual number of offspring of individuals with a minimum reproductive tenure of 1 year was lower in females (0.46 ± 0.33; range = 0 to 1.82; *n* = 268) than in males (0.74 ± 0.61; range = 0 to 2.83; *n* = 188; Wilcoxon rank-sum test: *W* = 18,695, *P* < 0.001). The total number of offspring was also lower in females (3.80 ± 3.71; range = 0 to 19; *n* = 287) than in males (4.95 ± 4.73; range = 0 to 26; *n* = 205; *W* = 25,817, *P* = 0.0196) (fig. S1). The higher mean values in males reflect the fact that fewer males than females successfully initiated a reproductive career. A total of 22.3% of females (*n* = 64) and 18.5% of males (*n* = 38) did not produce offspring during their reproductive tenure. On average, female reproductive tenure exceeded male reproductive tenure by 10.3 months (see Supplementary Text), consistent with the longer time males require to effectively start their reproductive career (see Materials and Methods). The mean number of grandoffspring was also lower for females (8.23 ± 15.63; range = 0 to 106; *n* = 287) than for males (11.29 ± 15.77; range = 0 to 85; *n* = 205; *W* = 24,236, *P* < 0.001) (fig. S2). A total of 47% of females (*n* = 135) and 33.7% of males (*n* = 69) had no grandoffspring. The mean number of great-grandoffspring was also lower for females (11.7 ± 26.0; range = 0 to 152; *n* = 168) than for males (16.5 ± 27.8.; range = 0 to 155; *n* = 123; *W* = 8690, *P* = 0.013) (fig. S3). A total of 56.5% of females (*n* = 95) and 43.1% of males (*n* = 53) had no great-grandoffspring.

### Reproductive inequality increases more in females across generations

**Fig. 1.**
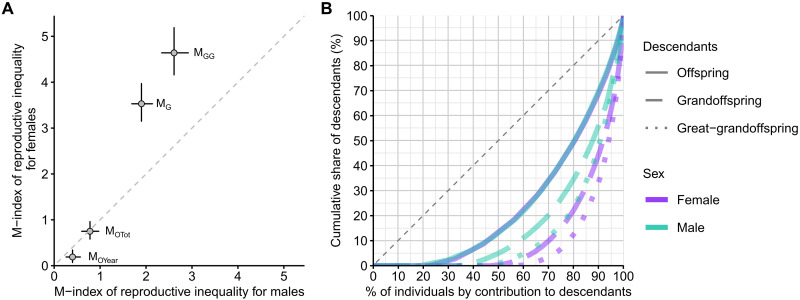
Comparison of reproductive inequality in female and male spotted hyenas. (**A**) Relationship between male (*x* axis) and female (*y* axis) M-indices of reproductive inequality for four measures of reproductive success: Annual number of offspring (M_OYear_), total number of offspring (M_OTot_), grandoffspring (M_G_), and great-grandoffspring (M_GG_). Filled circles are point estimates; horizontal and vertical lines around the circles indicate 95% credible intervals. The dashed diagonal line represents equal M-indices in the two sexes. (**B**) Lorenz curves depicting the cumulative distribution of descendant production for females and males, across three generations: offspring, grandoffspring, and great-grandoffspring. For each sex, individuals were ordered by their contribution to descendant production, and cumulative percentages were computed to visualize reproductive inequality. Male and female curves overlap at the offspring generation.

To quantify reproductive inequality in our four measures of reproductive success, we used the multinomial index (M-index) (*42*). This index compares observed reproductive success to that expected under an equal reproductive rate. Reproductive inequality was (i) lower in females than males for the annual number of offspring produced (M_OYear_), (ii) similar in females and males for the total number of offspring (M_OTot_), and (iii) higher in females than males for both the number of grandoffspring (M_G_) and great-grandoffspring (M_GG_) produced (Table 1 and Fig. 1A). To minimize the potential impact of the temporal variation in the ecological or demographic environment, we divided the study period into four cohorts and calculated separate M-indices for each cohort (fig. S4). The results were consistent with our main findings, with females showing higher reproductive inequality at the multigenerational level in each cohort, although the generation at which the sex reversal first became detectable varied.

**Table 1.** M-indices and Gini coefficients of reproductive inequality in female and male spotted hyenas. Annual offspring rates were calculated for all individuals with reproductive tenure greater than 0.1 year (M-index) or greater than 1 year (Gini coefficient), respectively. The three other measures represent total numbers of descendants. M-indices correspond to those depicted in Fig. 1A.

Measure	M-index female (95% CrI)^*^	M-index male (95% CrI)^*^	Gini female (95% CI)^†^	Gini male (95% CI)^†^
Offspring annual rate	0.19 (0.07–0.32)	0.41 (0.26–0.58)	0.4 (0.36–0.44)	0.45 (0.41–0.49)
Offspring total	0.76 (0.58–0.95)	0.78 (0.61–0.99)	0.52 (0.48–0.56)	0.51 (0.48–0.56)
Grandoffspring	3.53 (3.14–3.96)	1.91 (1.69–2.14)	0.76 (0.72–0.8)	0.66 (0.62–0.71)
Great-grandoffspring	4.63 (4.17–5.16)	2.62 (2.33–2.95)	0.83 (0.78–0.87)	0.74 (0.69–0.8)

* Values are posterior means and 95% credible intervals (CrI).

† Values are coefficients with 95% confidence intervals (CI) based on 1000 bootstrap replicates.

For comparison with previous studies, we also calculated Gini coefficients (Table 1) and plotted the corresponding Lorenz curves (Fig. 1B), which illustrate how reproductive success is distributed across individuals in each sex within the population (*43*). The top 19.49% of females and 19.8% of males, respectively, accounted for 50% of all offspring, indicating similar inequality. In contrast, for grandoffspring, the top 8.22% of females and 12.4% of males, respectively, produced half of all descendants, and for great-grandoffspring, these values declined further to 6.01% of females and 9.44% of males. Thus, reproductive inequality increased across generations in both sexes, but the increase was more pronounced in females than in males, reversing the sex bias in inequality from male to female (Fig. 1).

### Stronger intergenerational transmission of reproductive success in females

**Fig. 2.**
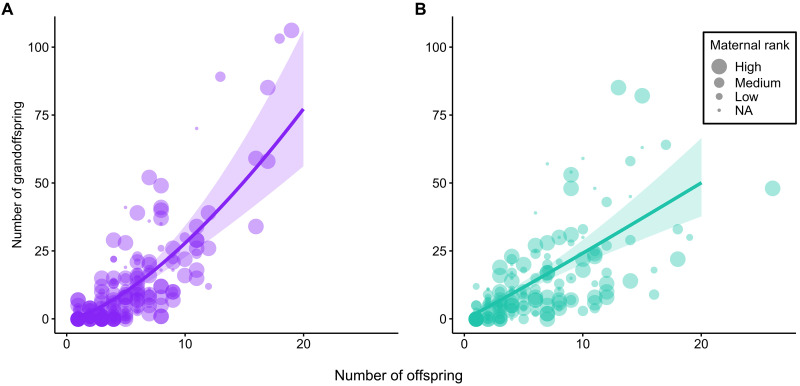
Relationship between the number of offspring and the number of grandoffspring produced by female and male spotted hyenas. Panels show the number of grandoffspring as a function of the number of offspring in (**A**) females (*n* = 223) and (**B**) males (*n* = 167); only individuals who produced at least one offspring are included. Symbols represent individuals, sized by maternal standardized social rank (high, medium, and low; NA, rank not assigned). Solid lines show model-predicted mean numbers of grandoffspring from zero-inflated negative binomial models, with shaded areas indicating 95% confidence intervals. Predictions are displayed on the original data scale.

To clarify why reproductive inequality increases more strongly in females compared to males, we examined the relationship between the number of offspring and the number of grandoffspring in the two sexes. We found that the (log) number of grandoffspring increased more strongly with the (log) number of offspring in females than in males (zero-inflated negative binomial model; χ^2^ = 8.32, df = 1, *P* = 0.00332; Fig. 2 and table S1).

Among individuals producing a large number of offspring, the correlation between the numbers of offspring and grandoffspring was particularly strong in females (e.g., for individuals producing 10 or more offspring: Spearman’s ρ_female_ = 0.679 versus ρ_male_ = 0.313; Fisher’s *z* test for correlation: *z* = 1.70; *P* = 0.0445; *n*_female_ = 21; *n*_male_ = 34). This result reflects the higher proportion of individuals born to mothers of high social rank (for individuals producing 10 or more offspring: 71.4% of females versus 41.2% of males were born from high-ranking mothers; see Fig. 2).

### Number of descendants of high-ranking mothers increases more along maternal than paternal lineage across generations

**Fig. 3.**
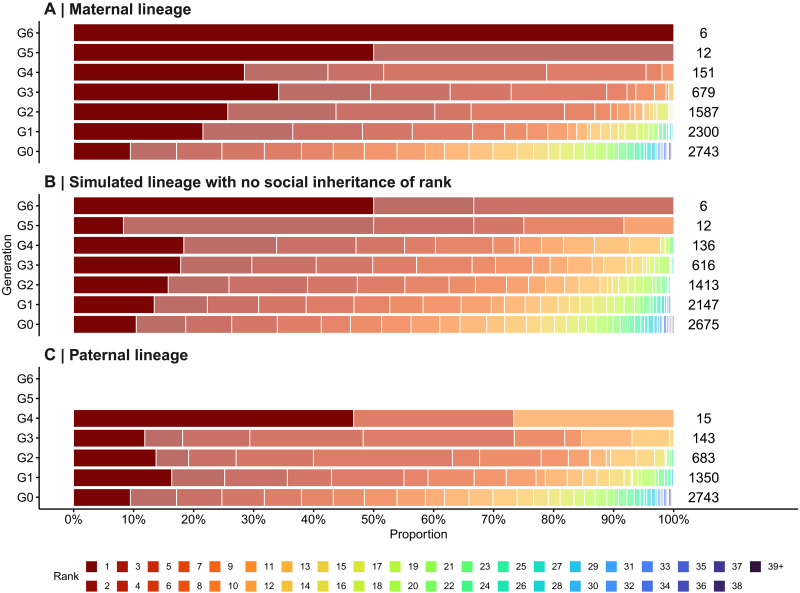
Effect of social rank inheritance: Distribution of social ranks among ancestors in maternal and paternal lineages. Stacked bars represent the distribution of ordinal ranks of ancestors within a given generation; ranks 39 and lower were pooled. Generation 0 (G0) contains all individuals with known ancestry—before tracing either maternal or paternal ancestral lineages. For the maternal lineage (**A**), the plot shows the maternal ranks of the mothers (G1), maternal grandmothers (G2), and so on of the individuals of G0. In (**B**), data were generated by permutation of ancestor identities from (A) under a scenario with rank-related reproduction but no social inheritance of rank. For the paternal lineage (**C**), the plot tracks the maternal ranks of the fathers (G1), paternal grandfathers (G2), and so on of the individuals of G0. Red colors indicate higher ranks, and blue colors indicate lower ranks. Numbers on the right of the bars are the numbers of individuals.

To explore the long-term, population-level effects of sex-biased rank inheritance, we quantified the distribution of ordinal ranks across maternal and paternal ancestors (Fig. 3). We calculated the maternal rank of all individuals with known ancestry (*n* = 2743) or “generation zero” (G0). We then reconstructed genealogical lineages backward in time, identifying the maternal rank of their mother (G1), their maternal grandmother (G2), and so on. For females, this approach captured the direct matriline; for males, we traced the maternal rank of each paternal ancestor (i.e., the father, the father of the father...).

Among females, the proportion of individuals born to top-ranking “alpha” mothers (see darkest red in Fig. 3A) increased markedly across generations traced backward from G0. In G0, only 9.48% of individuals were born to alpha mothers, compared to 21.6% in G1, 25.7% in G2, 34.2% in G3, and 28.5% in G4. Across these five generations, the proportion of alpha-born females increased 1.32-fold per generation on average. Among males, the proportion of individuals born to alpha mothers showed only a modest, nonmonotonic change from G0 to G3 (Fig. 3C). Although the proportion increased from 9.48% in G0 to 16.4% in G1, it declined in subsequent generations to 13.8% in G2 and 11.9% in G3, yielding overall a net change of 1.07-fold. Generations G5 and G6 for females, and G4 for males, were excluded from the fold-change analysis due to the small sample size (*n* < 20 individuals).

### Social inheritance increases the prevalence of top-ranking females among individuals’ ancestors

To test whether social rank inheritance increases the proportion of direct descendants of alpha females over generations, we conducted a permutation analysis that disrupted lineage continuity while preserving rank-related production of descendants. For each focal individual, we reassigned maternal identity by randomly selecting a mother from the same clan who had produced an offspring in the 2 years preceding the focal individual’s birth. This procedure removed social rank inheritance but retained the empirical variation in maternal reproductive success as high-ranking females produce more offspring and are more likely to be assigned as mothers. Across 1000 permuted datasets, the mean geometric fold-change in alpha rank frequency was 1.15 (see example in Fig. 3B), while the observed value in the maternal lineage was 1.32. No simulated values were equal to, or greater than, the observed values, yielding a permutation *P* < 0.001 (see fig. S4). Thus, the increase in the proportion of alpha-born ancestors when going backward in time was significantly greater than expected under the null model assuming no rank inheritance. These results demonstrate that social inheritance leads to descendants of alpha females becoming increasingly overrepresented across generations, beyond what would be expected from their higher reproductive success alone (simulated fold-change = 1.15 versus observed = 1.32).

### Highest share of descendants for alpha females

To evaluate how privilege translates into long-term reproductive success, we determined the social rank of the most successful females, defined as the females with the largest number of living descendants as of 1 January 2023, across the history of each clan (Table 2). Within each clan, most native individuals (between 58 and 100% of clan members) were descendants of the most successful females, all of which had held the alpha position during some period of their reproductive tenure. Those females were also ancestors of many descendants beyond their natal clan.

**Table 2.** Alpha status and number of direct descendants of the most successful female spotted hyenas in the eight Ngorongoro Crater clans. All descendant counts and clan characteristics were calculated as of 1 January 2023.

Females			Descendants				Clan		
			In population		In natal clan				
Name	Alpha^*^	Death year	Total	Alive	Alive	%^†^	Name	Natives	Total
A-013	Yes	2011	566	177	61	68.5	Airstrip	89	102
E-004	Yes	2002	715	164	32	82.1	Engitati	39	50
F-001	Yes	2002	590	174	31	93.9	Forest	33	40
L-007	Yes	1996	1267	283	37	97.4	Lemala	38	51
M-003	Yes	1996	1052	243	52	100.0	Munge	52	61
N-005	Yes	2002	577	140	20	62.5	Ngoitokitok	32	35
S-105	Yes	2013	219	51	43	79.6	Shamba	54	63
T-014	Yes	2016	89	31	15	57.7	Triangle	26	33

* Whether a female ever held the alpha position in the clan.

† Proportion of a female’s descendants born in and still alive in her natal clan at the end of the study period, relative to the total number of native individuals alive in that clan.

### Grandoffspring number better predicts long-term genetic contribution than offspring number in both sexes

**Fig. 4.**
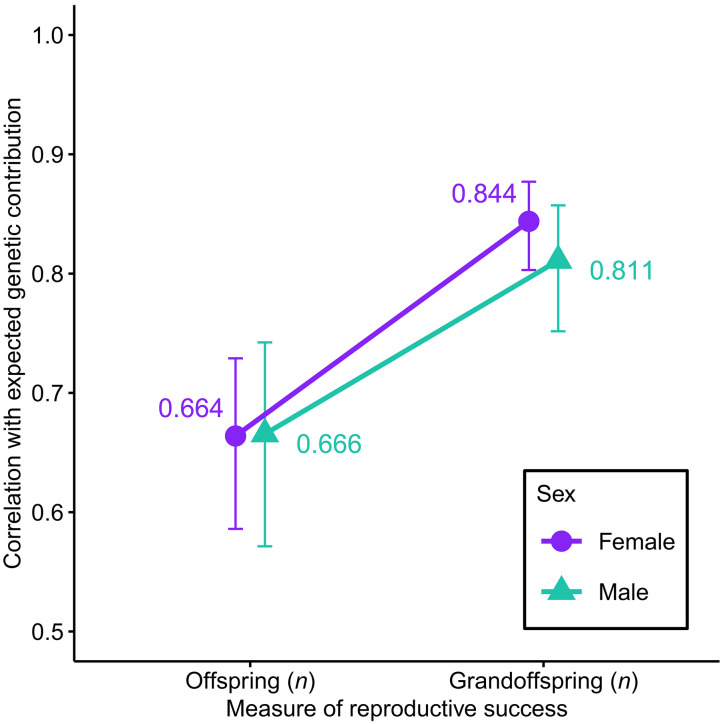
Correlation between offspring or grandoffspring number and expected genetic contribution in female and male spotted hyenas. Spearman’s correlation between expected genetic contribution (*g_i_*) and number of offspring or grandoffspring, shown separately for females (*n* = 239; in violet) and males (*n* = 167; in green). Points represent correlation coefficients, and error bars show 95% confidence intervals. Lines connect values within each sex to illustrate how the correlation with expected genetic contribution strengthens from offspring to grandoffspring counts.

Given that social inheritance amplifies reproductive inequality over generations, we next asked whether the number of grandoffspring is a better predictor of long-term genetic contribution than the number of offspring. For this, we used the population pedigree to quantify the expected number of copies of a new genetic variant ($g_{i}$) that an individual *i* would contribute to the population at the end of the study period (1 January 2023) (table S2). This revealed that $g_{i}$ was significantly more strongly correlated with the number of grandoffspring than with the number of offspring in both females (Fisher’s *z* test for correlation: *z* = −4.72; *P* < 0.001; *n* = 239) and males (*z* = −2.96; *P* = 0.00153; *n* = 167) (Fig. 4).

## DISCUSSION

Reproductive inequality can arise not only from variation in genetically heritable traits that influence reproductive success but also from nongenetic mechanisms such as the social inheritance of privilege. While the contribution of genetic inheritance to reproductive inequality has been investigated in some detail (*44*–*46*), much less is known about the effect of social inheritance of privilege, especially in nonhuman animals. Here, we show that social inheritance of privilege can be a strong driver of reproductive inequality and that it can reverse typical sex differences in reproductive inequality when inheritance is biased toward one sex.

In our study population of spotted hyenas, reproductive inequality in terms of the number of offspring produced per year was greater in males than females. This is consistent with previous findings in another hyena population (*47*) as well as with the general pattern in polygynous human populations and nonhuman mammals (*7*). This difference disappeared when reproductive inequality is measured in terms of the total number of offspring produced during the reproductive tenure, likely reflecting both female short-term reproductive constraints (small litter size, and long gestation and lactation periods) and sex differences in social rank trajectories (e.g., females maintain their maternally inherited rank, whereas dispersing males lose it). This pattern—whereby sex differences in reproductive inequality are reduced or disappear when measured over their reproductive tenure—was also documented in other species (*31*).

Over longer timescales, reproductive inequality increased in both sexes, but more markedly in females than in males, as predicted for a system where privilege is socially inherited through the maternal lineage (*48*). Females with high reproductive success also produced reproductively successful offspring, as indicated by the stronger offspring-grandoffspring relationship observed in females than in males. This pattern and the sex reversal in multigenerational inequality cannot be attributed to sex differences in life history, since both female and male focal individuals produce offspring of both sexes who are subject to the same dispersal and tenure constraints. Our simulations demonstrate that social inheritance of privilege, rather than rank-related reproductive output alone, led to descendants of the highest ranking females being strongly overrepresented in the population after only a few generations. The finding that rank inheritance—rather than rank-correlated fitness benefits alone—drives this pattern is conceptually important: It demonstrates that social inheritance of privilege operates as an evolutionary force in its own right. Our permutation analysis may not have fully disentangled rank inheritance per se from other correlated matrilineal advantages, but this should not strongly affect our findings because rank is the primary determinant of almost all social and ecological factors shaping the life of spotted hyenas (*32*, *34*, *36*, *49*), and the broad ecological environment, such as the territory and climate, is the same for all members of the clan, irrespective of their rank or matriline. Reproductive inequality is likely both the result and a driver of rank-related privilege. Since high-ranking females recruit more female offspring into their own clan, and female offspring provide kin support that consolidates high status (*50*), privilege also results from reproductive inequality. Both privilege and reproductive inequality are thus part of the same positive feedback loop (*13*, *47*).

In our study, sex-biased social inheritance leads to a notable sex reversal in reproductive inequality: While males showed greater inequality in short-term reproductive success, consistent with the predictions of the Darwin-Bateman paradigm, inequality became greater in females when grandoffspring were considered. Such female-biased reproductive inequality, albeit at the annual or lifetime level, was previously observed in species in which parental care is male-biased (*8*) or in which reproduction is monopolized by only one or a few females (*11*, *51*, *52*). Our results extend this observation by showing that even when females are the primary parental care givers and no particular female monopolizes reproduction, social inheritance can lead to a female bias in reproductive inequality provided that reproduction is measured in terms of descendants in future generations.

Female-biased reproductive inequality that concentrates reproductive success in a few high-ranking females, as observed in this study, is likely to intensify female-female competition (*12*, *53*). In species with male-biased parental care, females compete for access to caring males (*8*), and in cooperative breeders, females compete for breeding opportunities (*11*, *12*). What, then, do females compete for in systems shaped by social inheritance of privilege? Our finding that sex-biased reproductive inequality results from a stronger relationship between offspring and grandoffspring numbers suggests that females should try to secure and maintain a high social rank that they can pass on to their offspring along with the rank-associated privileges. In line with this idea, female hyenas fight directly over rank, rank reversals or “coups” do happen (*54*), and female infanticide—a hallmark of female-female competition across species (*55*)—is a leading cause of juvenile mortality (*56*) as well as a behavior that reduces the social support of females whose offspring are killed (*50*). Our findings contribute to a growing body of work emphasizing that competition among females is widespread and can exert strong selection (*53*, *57*–*59*), challenging the traditional view that intrasexual competition is predominantly a male phenomenon expressed through male-male competition for access to seemingly passive females (*60*).

Our findings further suggest that social inheritance of privilege reshapes optimal mating strategies in males and affects competition among them: Males could achieve higher long-term fitness by targeting high-ranking females, rather than by maximizing their number of matings or mating partners. Consistent with this, high-ranking male spotted hyenas allocate more courtship effort toward high-ranking females and sire more offspring with them than low-ranking males do (*41*, *61*). Those offspring, in turn, will also produce more offspring due to their higher maternal rank.

In systems with social inheritance of privilege, the number of grandoffspring is a more evolutionarily meaningful measure of fitness, as it captures both the quantity of offspring and their reproductive success—shaped by inherited privilege. Consistent with this, grandoffspring number predicted long-term genetic contribution more strongly than offspring number in both sexes, suggesting that grandoffspring counts are particularly informative in such systems—not only for quantifying reproductive inequality, but for evaluating which traits or behaviors meaningfully affect fitness.

One consequence of social inheritance of privilege through the maternal lineage is that high-ranking females ultimately contribute much more to the gene pool than low-ranking females. By hitchhiking on the positive, multigenerational, cascading effect of privilege, the alleles of males mating with privileged females can also reach high frequencies after a few generations. More generally, by increasing the variance in reproductive success, social inheritance of privilege can have far-reaching repercussions on genetic structure [for a review, see (*62*)]. In our study population, many individuals—particularly females—had no descendants, while a few highly successful females contributed disproportionately to the gene pool [although the genetic contribution of any ancestor inevitably gets diluted over generations (*63*)]. This is consistent with a qualitative pattern described in a spotted hyena clan of a different population: 30 years after the onset of their study, adult female descendants of the initial alpha and beta females comprised 80% of all adult females in the clan (*47*). Similar patterns of extreme lineage loss driven by high reproductive variance and heritable differences in fitness were documented in other vertebrates such as cheetahs (*Acinonyx jubatus*) (*64*).

When reproduction is concentrated in a small subset of individuals, effective population size declines, and this can decrease the efficiency of natural selection (*65*–*67*), accelerate the loss of genetic diversity (*68*), and elevate inbreeding (*65*). Certain behaviors may buffer populations against the genetic risks brought by the social inheritance of privilege. In spotted hyenas, male-biased dispersal (*40*, *69*), female mate preferences (*40*), and female promiscuity (*70*) are likely to increase gene flow and reduce inbreeding. Whether such behaviors are sufficient to counter the negative long-term genetic effects and whether they are required for the social inheritance of privilege to evolve or are an adaptive response to the presence of such systems remain open questions.

Given its potential impact upon within- and between-sex competition and genetic evolution, an important question is how common social inheritance of privilege is. Evidence from both human and animal societies suggests that social inheritance of privilege is not unique to spotted hyenas. In humans, privilege can take the form of land, livestock, economic resources, or social status, all of which influence access to mates, marriage opportunities, and reproductive outcomes across generations (*15*, *30*). For example, among the Gabbra pastoralists, livestock is inherited patrilineally and unequally among sons, and it is a key determinant of marriage opportunities and reproductive success (*71*). In animals, socially inherited privilege can take many forms, including territories, stored resources, shelter, and social status, with documented examples across a range of taxa (*13*, *14*). The impact of social inheritance on reproductive inequality likely depends on privileges being retained within the lineage over multiple generations. When this condition is met, inequality can intensify across generations, and do so to an extent shaped by how strongly privileges influence fitness. We thus expect that many species have intrasexual competition and genetic evolution shaped by the social inheritance of privilege. In particular, this should be the case in species where social conventions make inheritance consistent across generations.

For social inheritance of privilege to generate a sex reversal in reproductive inequality, as shown here for spotted hyenas, privilege must (i) be transmitted through the sex that shows lower reproductive inequality at the single generation level, and (ii) have a strong effect on reproductive success. These conditions are more restrictive than those required for social inheritance of social privilege alone, yet several species are likely to qualify. In particular, cercopithecines seem good candidates because, in many of these primates, rank is maternally inherited (*29*, *72*) and high rank increases fitness-related traits such as lifetime reproductive success [in long-tailed macaques *Macaca fascicularis* (*73*) and yellow baboons *Papio cynocephalus* (*74*)] and infant survival [in vervet monkeys *Chlorocebus pygerythrus* (*75*)]. Similar processes may also operate in human societies with matrilineal inheritance systems, and recent cross-cultural analyses show wide variation in the direction of wealth transmission rather than a universal male bias (*27*). Detecting such effects requires datasets spanning several generations—data that are currently scarce for wild populations. Long-term studies of social species with known pedigrees will therefore be essential, alongside theoretical models, to test how different inheritance systems generate reproductive inequality across species and contexts.

Future studies should explicitly consider social inheritance of privilege. Social inheritance of privilege shares key features with genetic inheritance (both can show substantial heritability, influence fitness variation, be sex-biased, and exert large consequences on the gene pool), but important differences remain. For example, unlike genetic transmission—where all offspring have equal probability of inheriting alleles that enhance access to resources—social privilege is typically restricted to particular offspring [e.g., the eldest son in many human societies (*76*); the youngest in spotted hyenas and cercopithecines (*16*, *29*, *33*)]. The social inheritance of privilege also recalls cultural inheritance since the transmission of privileges across generations is not genetic (*16*). However, contrary to cultural traits, privilege is based on social conventions that seem to vary little across time and space: All spotted hyena clans that have been studied to date show linear dominance hierarchies and the same mechanisms of rank inheritance.

Our findings have broad implications for the Darwin-Bateman paradigm. For spotted hyenas, the paradigm predicts higher reproductive inequality in males and stronger male-male than female-female competition, which would be traditionally interpreted as selecting for male-biased morphological dimorphism. However, spotted hyenas show a striking reversal of this prediction. Females are as large as males (*77*), engage in intense female-female competition (*56*), and therefore appear to be the more competitive sex. This inconsistency has been noted as puzzling in the mammalian literature (*11*).

We propose a previously unidentified mechanism that can explain the deviation from the pattern of inequality predicted from anisogamy alone. Social inheritance of privilege can generate female-biased reproductive inequality gradually across generations through the compounding effects of maternal rank inheritance. This does not invalidate the core logic of the Darwin-Bateman paradigm—rather, it reveals that the paradigm’s predictive power is partially restored once reproductive inequality is measured at the appropriate timescale. The paradigm correctly predicts that the sex with higher reproductive inequality will be the more competitive one. In species where social inheritance of privilege operates, a single generation of reproductive data can be insufficient to reveal which sex faces stronger reproductive competition.

In conclusion, our study provides strong evidence that the social inheritance of privilege can shape reproductive inequality and reverse its typical sex bias. Our study also contributes to the growing recognition that social inheritance has far-reaching genetic and evolutionary consequences (*48*, *78*). Together, these findings suggest that the conditions under which females become the more unequal and more competitive sex are broader than previously recognized, and that incorporating social inheritance into the Darwin-Bateman framework may be essential for explaining the diversity of sex differences observed across animal societies.

## MATERIALS AND METHODS

### Study species and population

Spotted hyenas live in social groups called clans, which have linear dominance hierarchies and a high level of female dominance (*50*). Offspring of both sexes acquire a rank just below their mother through behavioral mechanisms of social learning and maternal social support (*16*, *28*). Accordingly, offspring resemble mothers in social ties, especially in high-ranking lineages (*18*). Female hyenas typically produce one or two cubs per litter (rarely three), with a lactation period ranging from 6.7 to 18.8 months and an interbirth interval ranging from 6.6 to 28.7 months, limiting their overall reproductive output (*34*, *38*, *79*, *80*). Reproduction does not follow a marked seasonal pattern, so generations overlap (*34*). Dispersal is strongly male-biased with ∼85% of males leaving their natal clan before reproducing, and immigrating to a new clan to breed, whereas females are highly philopatric (*39*–*41*). In their new clan, immigrant males lack social support and enter at the bottom of the hierarchy. They progressively increase in rank over time, and while they may eventually reach a high rank among immigrants, they will remain lower ranking than native individuals (*50*, *81*, *82*). High-born males do retain their maternal rank and benefit from priority access to resources during excursions to the territory of their natal clan, but these extended benefits only last up to 2 years after dispersal (*39*).

We studied the spotted hyenas of the eight resident clans of the 300-km^2^ Ngorongoro Crater in Tanzania (3°11′S, 35°34′E) between 12 April 1996 and 21 February 2025. All individuals were individually identified by their unique spot pattern and other individual morphological features (e.g., ear notches). Most males (>83%) reaching the age of clan choice and born in one of the Crater clans choose to breed in a Crater clan (*41*), which allowed us to record the reproductive output of philopatric and dispersing males and to compare females and males. We classified females as reproductively active from 2 years of age onward, as high-ranking females typically start reproducing at that age (*32*). In the rare cases when a female conceived before the age of 2 years, her reproductive career was considered to have started from the date of conception. Dispersing males were considered reproductively active from their first confirmed sighting in a new clan’s territory followed by sustained presence and social integration (marking the start of their tenure). For philopatric males, we considered them as reproductively active from the date they first displayed sexual interest in females and continued doing so for at least three consecutive months (*41*). The average age at the onset of reproductive tenure was 2.0 ± 0.02 years in females and 3.38 ± 0.76 years in males. This reflects the longer time males require entering the breeding pool. The delayed expression of sexual behaviors in males is also reflected in reproductive data: The median age at first conception (estimated as offspring birthdate minus 110 days of gestation) was 3.62 years in females [interquartile range (IQR) = 2.9 to 4.3, *n* = 239) and 5.28 years in males (IQR = 4.2 to 6.3, *n* = 167). Reproductive tenure was defined as the time from the estimated onset of reproductive activity until the date of death or the end of the study, whichever came first. All fieldwork and research protocols were approved (no. 2023-01-03) by the Internal Committee for Ethics and Animal Welfare of the Leibniz-IZW.

### Sample collection and paternity analysis

Biological samples such as tissue, hair, and feces, were collected opportunistically. Fecal samples consisted of the epithelial-rich mucus layer surrounding fresh feces, collected directly after defecation. All biological material was preserved in either ethanol or a dimethyl sulfoxide salt solution until DNA extraction. Parentage was determined using nine highly polymorphic microsatellite loci (*83*) with a mean of 11.9 alleles per locus (range: 7 to 16) and a mean expected heterozygosity of 0.83. Paternity was assigned using maximum likelihood methods as implemented in CERVUS 3.0 (*84*) based on candidate fathers present in the natal clan at the estimated date of conception. Total exclusionary power was 0.999 and the error rate was 0.44% and set at 1.0%. Paternity was assigned at the 95% confidence level. For full methodological details, see (*41*) and references therein.

### Data analysis

All analyses were conducted in R (version 4.5.0). Data were compiled into a centralized database and processed using the hyenaR package version 0.10.0.9000 (*85*). Summary statistics correspond to means ± SD, unless stated otherwise. For the statistical tests, significance was assessed at the threshold α = 0.05. We fitted all statistical models with the package glmmTMB version 1.1.9 (*86*). Model diagnostics were assessed using package DHARMa version 0.4.7 (*87*). ChatGPT (OpenAI), specifically GPT-4o and GPT-5, was used to assist in improving and debugging code during data analysis; all code and results were independently verified by the authors.

### Reproductive success

We quantified reproductive success as (i) the mean number of offspring produced per year during the reproductive tenure, (ii) the total number of offspring produced during the reproductive tenure, and the total number of (iii) grandoffspring and (iv) great-grandoffspring produced. Reproductive success was assessed based on genetically confirmed parent-offspring relationships, using only cubs for which both maternity and paternity were verified through genetic analysis. This ensured that our estimates of reproductive success were evolutionarily relevant and comparable between mothers and fathers, and minimized potential biases from adoptions, which are rare in our population (see below). Grandoffspring and great-grandoffspring were included only if they descended from such genetically confirmed offspring, ensuring consistent criteria across generations and between sexes. Mean annual reproductive success was calculated by dividing the total number of offspring by the reproductive tenure. To avoid inflated estimates due to small denominators, we included only individuals with a reproductive tenure greater than 1 year in the mean number of offspring produced per year. To ensure accurate and comprehensive estimation of the total number of offspring, we included only individuals who (i) were born in a study clan, (ii) initiated their reproductive career between 12 April 1996 and 1 January 2023 (the onset of monitoring of our study population and the study end date for analyses requiring complete genetic information, respectively), (iii) had not been recorded to ever disperse to a clan outside the study area, and (iv) had been genetically typed for parentage analyses. Our data comprised 1187 known reproductively active individuals. We excluded 123 individuals not born in a study clan, 66 who initiated reproduction before the beginning of the study, 19 who dispersed to a clan outside the study area, and 16 without a genetic sample. We assigned a cohort for each individual based on the year in which they initiated their reproductive career. We computed all measures of reproductive success for all individuals who initiated their reproductive career before 1 January 2011 (with the additional tenure-criterion mentioned above for annual reproduction estimates). Individuals initiating their reproductive career during the year 2010 constituted the most recent yearly cohort for which at least 95% of individuals had died by 1 January 2023, ensuring near-complete reproductive histories. The resulting sample included 456 potential parents with a minimum tenure of 1 year, included in a larger set of 492 individuals with complete or near-complete reproductive tenures. Within this later set, 487 (98.98%) had an estimated death date by 1 January 2023, and the remaining 1.02% had near-complete reproductive tenures. For the numbers of grandoffspring, 11.7% of offspring (150 of 1284) were still alive on 1 January 2023 and had not yet completed their reproductive careers. To ensure comparability between female and male reproductive success estimates and to evaluate the potential impact of censored data on grandoffspring counts, we also assessed the overall degree of completeness in grandoffspring counts and tested whether it differed between the sexes (see Supplementary Text). To ensure the reliability of analyses on great-grandoffspring counts, we restricted those particular analyses to individuals from cohorts with a mean completeness exceeding 0.95, corresponding to cohorts up to and including 2006. This yielded a reduced sample of 291 individuals (168 females and 123 males). Although absolute numbers should be interpreted with caution, great-grandoffspring counts remain suitable for female versus male comparisons because all focal individuals initiated their reproductive career within the same time window.

### Reproductive inequality

To quantify reproductive inequality, we first used the multinomial M-index (*42*). This index captures the extent to which reproductive success is unevenly distributed among a group of individuals. When all individuals are considered to have equal exposure time (e.g., the entire lifespan), the M-index reduces to the traditional metric *I* quantifying the opportunity for selection (*42*). The M-index is constructed to be robust to differences in sample size and mean reproductive success, enabling standardized comparisons across populations, sexes, and species. We calculated the M-index for different measures of reproductive success: (i) the number of offspring produced per year (M_OYear_), (ii) the total number of offspring (M_OTot_), (iii) the total number of grandoffspring (M_G_), and (iv) the total number of great-grandoffspring (M_GG_). M_GG_ was highly skewed, and to ensure numerical stability in the Bayesian estimation for this metric, we followed developer guidance for setting priors (mean = 8 ± 0.2) on the concentration term in their model and increased some of the Markov chain Monte Carlo tuning parameters (adapt_delta = 0.99, max_treedepth = 14). These adjustments minimized divergent transitions and improved convergence of the posterior estimates. All M-indices and their 95% credible intervals were calculated separately for females and males using the R package SkewCalc (*42*). Two females with reproductive tenure shorter than 0.1 years were excluded from the calculation of the M_OYear,_ as such short tenures were causing fitting issues. We also computed the Gini coefficient and relative confidence intervals (*88*) using the Gini function from the R package DescTools (version 0.99.60) (*89*) and represented their associated Lorenz curves for offspring, grandoffspring, and great-grandoffspring, separately for each sex. The Gini coefficient is a widely used metric to quantify inequality that is bounded between 0 (maximum equality) and 1 (maximum inequality). Confidence intervals for Gini coefficients were derived by bootstrapping (1000 replicates, bias-corrected accelerated method). For the Gini coefficient of the annual offspring rate, we applied the same minimum tenure criterion (>1 year) used for the mean annual reproductive success estimates. For each sex, individuals were sorted by their number of descendants, and cumulative proportions of individuals and reproductive output were used to construct the Lorenz curves (*43*).

M-indices and Gini coefficients were calculated at the population level, pooling all individuals irrespective of clan membership, ensuring that estimates of reproductive inequality are not influenced by sex-biased dispersal patterns. The M-index is mathematically similar to the Gini coefficient but, unlike the Gini coefficient, accounts for differences in reproductive tenure between individuals, making it a more robust measure of inequality (*42*).

To assess whether the sex reversal in inequality was robust to comparisons across different ecological and demographic contexts, we repeated the M-index calculations separately for four contemporaneous cohorts defined by the year individuals initiated their reproductive career: 1996–1999 (*n* = 41 females, 36 males), 2000–2003 (*n* = 79 females, 50 males), 2004–2007 (*n* = 80 females, 64 males), and 2008–2010 (*n* = 87 females, 55 males). Within each cohort, only individuals who initiated their reproductive career in the same time window were compared, ensuring the comparison of contemporaneous cohorts. For great-grandoffspring, analyses were further restricted to cohorts with mean completeness exceeding 0.95 (up to and including 2006). The first two cohorts (1996–1999 and 2000–2003) were unaffected, while the 2004–2007 cohort was reduced to (*n* = 48 females, *n* = 37 males), and the 2008–2010 cohort was excluded entirely as it falls outside the completeness threshold.

### Maternal dominance rank at birth

Dominance hierarchies were constructed based on observed outcomes of dyadic agonistic interactions among adults in the same clan at the beginning of the study. Rankings after this initial period were updated iteratively to reflect the most consistent linear order, based on conventions such as maternal rank inheritance and social queuing and behavioral observations. For full details, see (*61*). We defined maternal dominance rank at birth as the ordinal position of an individual’s mother in the adult female dominance hierarchy on the date of the individual’s birth. Because systematic dominance data began on 12 April 1996, for individuals born between 12 April 1995 and 12 April 1996 (i.e., alive as subadult at the start date of the project), we used their mother’s rank on 12 April 1996 as approximation for the unknown rank of their mother at birth; the same approximation was applied when assigning standardized ranks. For a few individuals who started their reproductive career after 12 April 1996 but born before 12 April 1995, we did not assign them maternal ranks. The maternal dominance rank at birth reflects the maternal social status an individual was born into and serves as a proxy for early-life social conditions and as a proxy for privilege. For brevity, we refer to this variable as maternal rank. For visualization purposes in Fig. 2, maternal rank was further divided into categories. Specifically, we calculated the standardized maternal dominance rank, which ranges from −1 (lowest) to +1 (highest), and partitioned this continuous measure into three categories: high (≥0.333), medium (−0.333 to <0.333), and low (<−0.333).

### Sex differences in the offspring-grandoffspring relationship

We fitted statistical models to investigate the relationship between the number of grandoffspring and the number of offspring across sexes. The response variable was the number of grandoffspring produced per individual. We modeled the expected number of grandoffspring as a function of the log-transformed number of offspring, data completeness, and sex, including interactions between sex and both predictors. The number of offspring was log-transformed to linearize the relationship. The variable completeness corresponds to the proportion of an individual’s offspring that were deceased by the end of the study. Completeness was included as a covariate to account for differences in the proportion of offspring with full record of reproductive success per individual, serving as an estimator of how complete the observed grandoffspring counts were. We *z*-transformed this variable to facilitate convergence during the fitting procedure. Individuals with zero offspring always have zero grandoffspring and were therefore excluded from this analysis.

Given the count nature of the data and an excess of zero values compared to Poisson expectation, we attempted to fit a series of models with different distributional assumptions: a zero-inflated Poisson generalized linear model (ZIP), a negative binomial model (NB2), and zero-inflated negative binomial models (ZINB2). All these models considered the log function as their link function. We retained the ZINB2 model as this was the only one for which modeling assumptions were satisfied (as evaluated using DHARMa). For zero-inflated models (including the retained ZINB2 one), we modeled the zero-inflation component (i.e., the logistic regression submodel) with either an intercept only or with an intercept and a linear effect of completeness, as more incomplete offspring records could increase the likelihood of zero grandoffspring. Including completeness in the formulas of the zero-inflation components improved model fits substantially, so we retained this formulation in the final ZINB2 we used. The significance of estimates was tested using a series of likelihood ratio test following a type II analysis of variance (ANOVA) design using the R package car (*90*), which internally calls the method implemented in glmmTMB. For Fig. 2, model predictions and their associated 95% confidence intervals were plotted on the response scale, corresponding to the expected number of grandoffspring for a given offspring number at mean completeness (i.e., *z* = 1).

To examine how the relationship between the number of grandoffspring and offspring varied between sexes at high reproductive output, we restricted the dataset to individuals that produced 10 or more offspring. For each sex, we calculated Spearman’s rank correlation coefficient (ρ) between the number of offspring and grandoffspring. Differences between female and male correlations were tested using the function diffcor.two from the R package diffcor (version 0.8.4) (*91*). To assess whether maternal rank contributed to observed sex differences, we also calculated for each sex, the proportion of individuals in the subset that were born to high-ranking mothers (standardized maternal rank category = high).

### Pedigree construction

We built a multigeneration pedigree for all study individuals using a combination of genetic parentage assignment and behavioral observations of suckling (consistent mother-cub associations during lactation).

### Intergenerational persistence of social rank: Maternal versus paternal lineage

To test whether sex-specific differences in the intergenerational persistence of maternal rank were associated with patterns of social rank inheritance, we reconstructed genealogical lineages using the pedigree for all individuals born during the study period and with at least one known parent. We identified a total of 2868 individuals. Of these, 2743 had a known mother. For maternal identity, we used the socially assigned mother since we were interested in the effect of the transmission of social ranks and since cubs inherit the ranks of their social mother. In 58 cases, the social and genetic mother differed, indicating instances of adoption. In addition, genetic data were available to assign paternity for 1839 individuals.

The 2743 individuals with a known mother were defined as focal individuals belonging to generation 0 (G0), irrespective of birth year. For each focal individual, we calculated the maternal rank of their mother (G1), maternal grandmother (G2), and earlier ancestors along the direct maternal (social) lineage. We also reconstructed an equivalent paternal lineage and identified the maternal rank of each paternal ancestor—i.e., the focal individual’s father (G1), paternal grandfather (G2), and so on. This approach allowed us to quantify and compare the intergenerational persistence of maternal rank across sexes. To avoid sparsity in the lower tail of the distribution of social ranks and facilitate comparisons across generations and sexes, we grouped individuals that were ranked 39th or below (40th, 41st...) according to their maternal ranks into one single ordinal class.

To quantify the intergenerational persistence of maternal rank among top-ranking (“alpha”) individuals, we calculated, for each generation, the proportion of individuals whose maternal ordinal rank was equal to one. We then measured the fold-change in the proportion of alpha-ranked ancestors between successive generations. A geometric mean of these fold-changes was used to obtain a single summary statistic across all generations including 20 individuals or more to ensure reliable and informative measures.

To test whether the observed persistence of maternal rank differed from expectations under a model with rank-related fitness benefit but without rank inheritance, we performed an analysis by permutation. For each ancestor in each generation, we reassigned maternal rank by randomly selecting a mother from the same social clan and cohort window (defined as the 2-year period preceding the birth of the focal ancestor). If the 2-year window extended before 12 April 1995, the corresponding ancestor was excluded from the permutation analysis since no maternal rank could be inferred with precision. This permutation scheme preserved the demographic and social structure of the population, including variation in reproductive output and the elevated reproductive success of high-ranking females, but disrupted lineage continuity by breaking the parent-offspring inheritance of rank. We repeated this permutation procedure across all generations and recalculated the geometric mean of fold-changes in the proportion of alpha individuals between successive generations, for each permutation round, to generate a null distribution of the summary statistic. As before, fold changes were computed only for generations with ≥20 eligible individuals. The observed geometric mean was then compared to this null distribution to evaluate whether the persistence of high maternal rank across generations exceeded expectations under the null model. The *P* value was calculated as the proportion of permuted values that were equal to or greater than the observed value, with a standard correction (*92*).

### Long-term genetic contribution

For each focal individual, we quantified their genetic contribution as the expected number of copies of their alleles present in the population as of 1 January 2023. For each focal individual *i*, we calculated *g_i_*, defined as the sum of pedigree-derived pairwise relatedness coefficients to all known descendants of *i* that were alive at that date

gi=∑j=1nrij (1)

where $r_{\mathrm{ij}}$ is the coefficient of relatedness between focal individual i and the descendant j as calculated from the pedigree, and n is the number of recorded descendants alive at the end date.

To examine how well simple reproductive counts capture genetic representation, we calculated Spearman correlations between $g_{i}$ and the number of offspring and grandoffspring (as of 1 January 2023) for each individual included in the reproductive inequality analysis. Because the pedigree used to count descendants incorporated behavioral observations, we also considered offspring whose mothers were assigned behaviorally or genetically without assigned fathers, relaxing the stricter criteria used in the reproductive-success analyses. Correlation analyses included only individuals with at least one offspring, resulting in a sample size of 406 individuals.

### Share of descendants for most successful females

We analyzed females with the highest number of living descendants as of 1 January 2023. This analysis included all adult females known during the study period. Using the pedigree of the population, we counted each female’s total number of unique descendants and determined which of those were alive at the target date. For each female, we recorded (i) the total number of descendants, (ii) the number of descendants alive, and (iii) the number of living descendants that were present in the female’s natal clan on 1 January 2023. We then identified, within each clan, the female with the highest number of living descendants. For each of these most successful females, we determined whether they ever reached the alpha position during their lifetime, and we recorded their year of death. To provide context on lineage persistence, we calculated the proportion of each female’s living descendants that remained in her natal clan relative to the total number of native individuals alive in that clan, regardless of whether their ancestry was fully known or not. Clan size estimated for 1 January 2023 is also reported and includes both native individuals and immigrant males.

## Supplementary Materials

### This PDF file includes:

Supplementary Text Figs. S1 to S5 Tables S1 and S2 References
