# Supplementary material for: The legacy of privilege: Social inheritance reverses sex differences in reproductive inequality in the spotted hyena
Source: Sci Adv. 2026 Jul 23;12(30):eaee7880. doi: 10.1126/sciadv.aee7880 (PMC13394369; doi:10.1126/sciadv.aee7880)
Supplement: Supplementary file 1 — Supplementary Text Figs. S1 to S5 Tables S1 and S2 References [file sciadv.aee7880_sm.pdf]

Science Advances
AAAS

Supplementary Materials for

The legacy of privilege: Social inheritance reverses sex differences in reproductive inequality in the spotted hyena

Marta Mosna *et al.*

Corresponding author: Marta Mosna, marta.mosna@evobio.eu, mosna@izw-berlin.de

Sci. Adv. **12**, eaee7880 (2026)
DOI: 10.1126/sciadv.aee7880

This PDF file includes:

Supplementary Text
Figs. S1 to S5
Tables S1 and S2
References

Supplementary text

Completeness of grandoffspring counts across sexes

Incomplete (censored) grandoffspring counts may underestimate multigenerational reproductive success. Because our reproductive success metrics included the number of grandoffspring, we evaluated how complete these counts were for each focal individual. We use the term "completeness" to refer to the degree to which an individual's grandoffspring count could be considered complete at the end of the study period. We considered counts to be complete when all the genotyped offspring of a focal individual had died—and thus had uncensored opportunities to reproduce. Individuals whose offspring were still alive and potentially reproducing beyond the study's end were considered having incomplete grandoffspring count.

To test for potential sex-specific biases in data completeness, we first compared the proportion of individuals with no surviving offspring between females and males using a Fisher exact test. Among individuals with at least one surviving offspring, we compared the number living offspring between sexes using a Wilcoxon rank-sum test. Finally, we calculated an individual-level completeness score, defined as the proportion of an individual's offspring that had deceased by the end of the study. Individuals with no offspring were assigned a score of 1.0 (fully complete).

Of all selected individuals (n = 492), 77.4% had no offspring alive at the end of the study period: 79.8% (n = 229 out of 287) of females and 74.1% of males (n = 152 out of 205). This difference was not significant (Fisher's exact test; odds ratio = 0.727, 95% CI: 0.465–1.14, p = 0.155). Among individuals with at least one living offspring (n = 111), the number of living offspring did not differ significantly between sexes (Wilcoxon rank-sum test: W = 1,363, p = 0.258). Completeness scores were generally high (mean = 0.932, SD = 0.162). Females (mean = 0.944, SD = 0.135) and males (mean = 0.914, SD = 0.192) completeness scores did not differ significantly (Wilcoxon rank-sum test: W = 31,220, p = 0.113).

Mean reproductive tenure in females and males

The mean reproductive tenure was nearly one year longer in females (7.31 ± 4.34 years; range = 0.002–16.46; n = 287) than in males (6.45 ± 3.66 years; range = 0.109–15.30; n = 205; Wilcoxon rank-sum test: W = 32,684 , p = 0.0357). Among individuals with a minimum tenure of one year, mean reproductive tenure was also significantly higher in females (7.80 ± 4.08 years; range = 1.07–16.46; n = 268) than in males (6.99 ± 3.36 years; range = 1.03–15.30; n = 188; Wilcoxon rank-sum test: W = 27,993, p = 0.043).

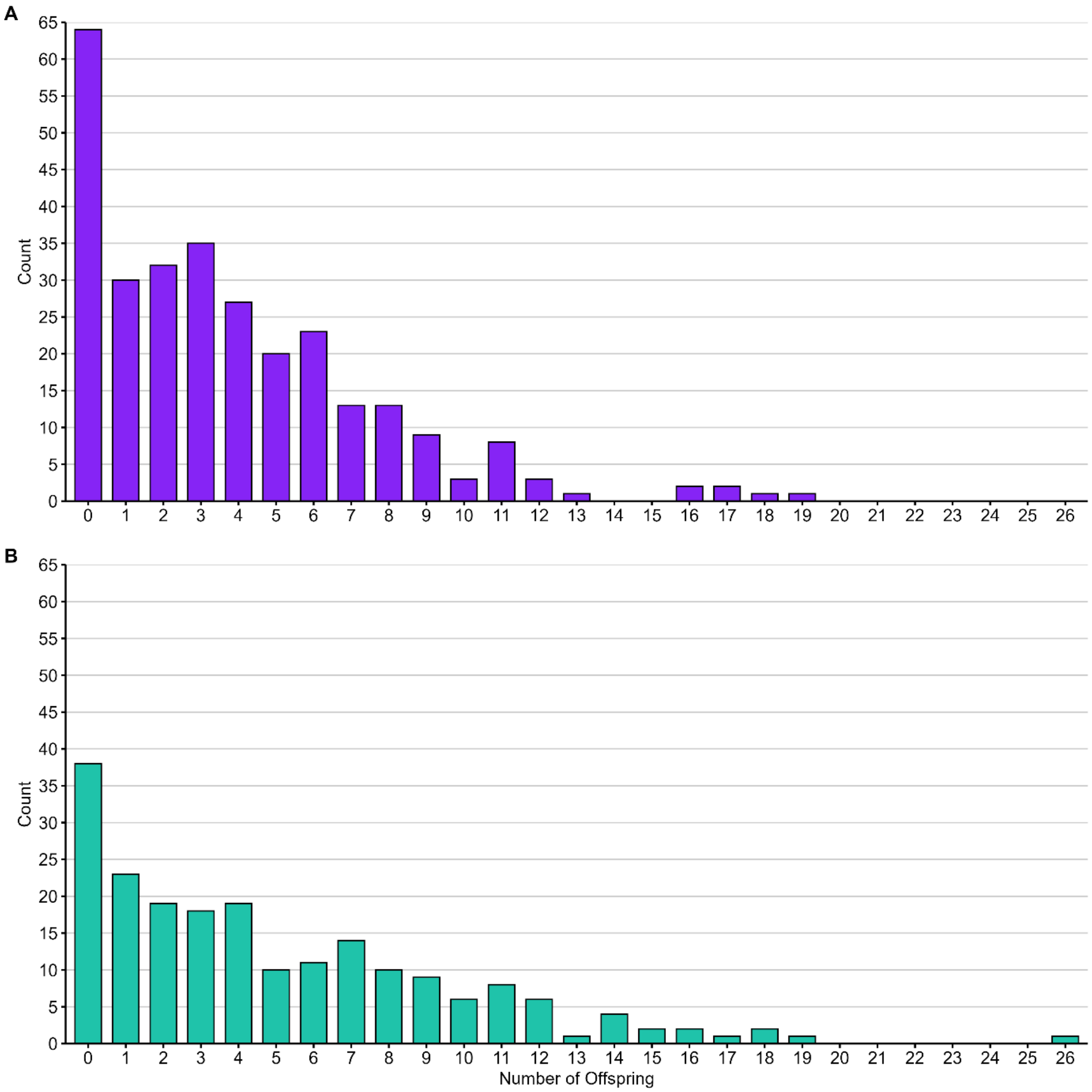

Fig. S1. Frequency distributions of lifetime reproductive success in female and male spotted hyenas.

Number of offspring produced (**A**) by females (n = 287, in violet) and (**B**) by males (n = 205, in green). Lifetime reproductive success was defined as the total number of offspring produced by each individual during their lifetime.

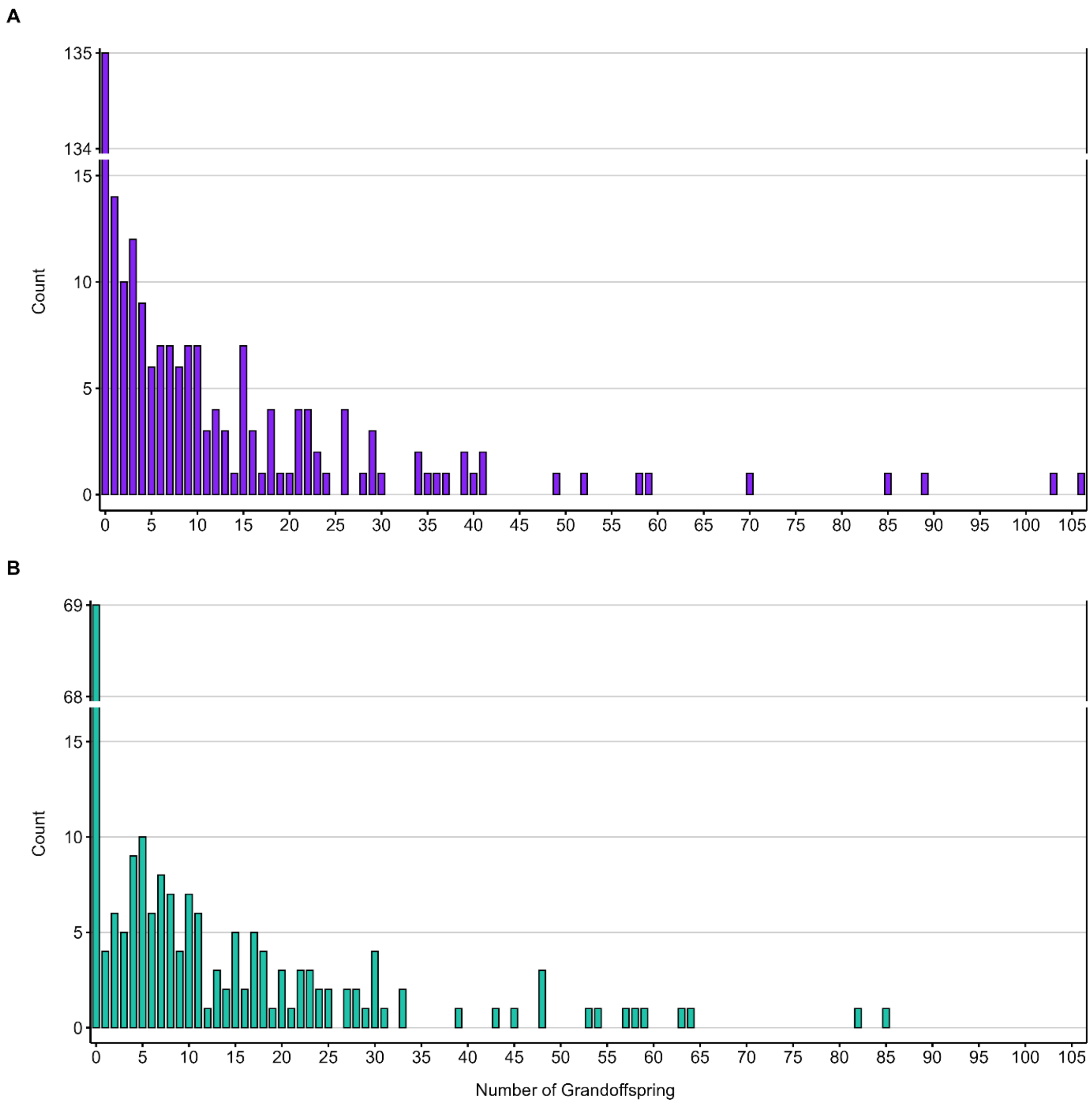

Fig. S2. Frequency distributions of grandoffspring counts in female and male spotted hyenas.

Number of grandoffspring produced (**A**) by females (n = 287, in violet) and (**B**) by males (n = 205, in green). Y-axis breaks were used to improve readability given the high frequency of individuals with zero grandoffspring (*93*).

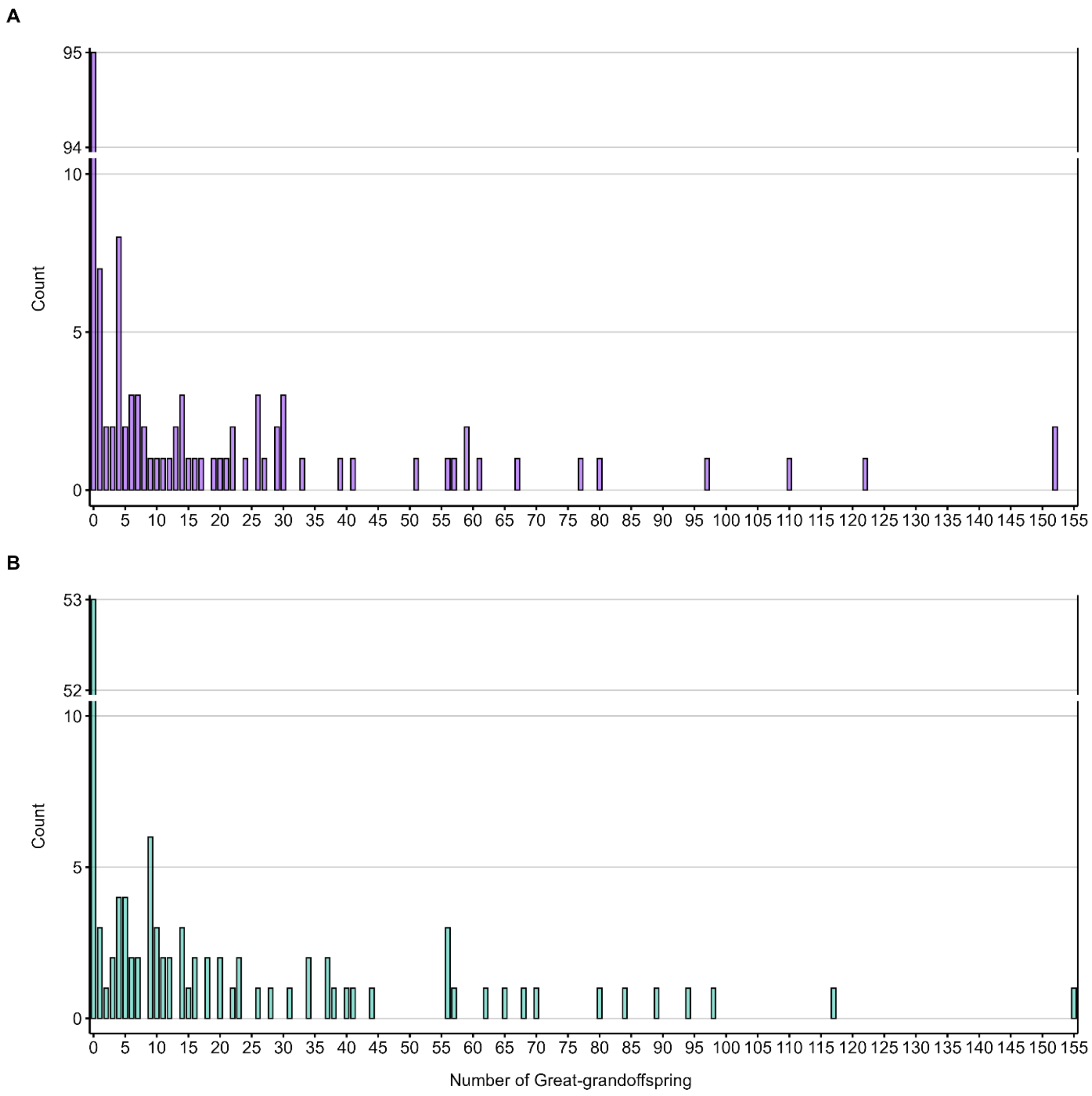

Fig. S3. Frequency distributions of great-grandoffspring counts in female and male spotted hyenas.

Number of great-grandoffspring produced (**A**) by females (n = 168, in violet) and (**B**) by males (n = 123, in green). Y-axis breaks were used to improve readability given the high frequency of individuals with zero great-grandoffspring (*93*).

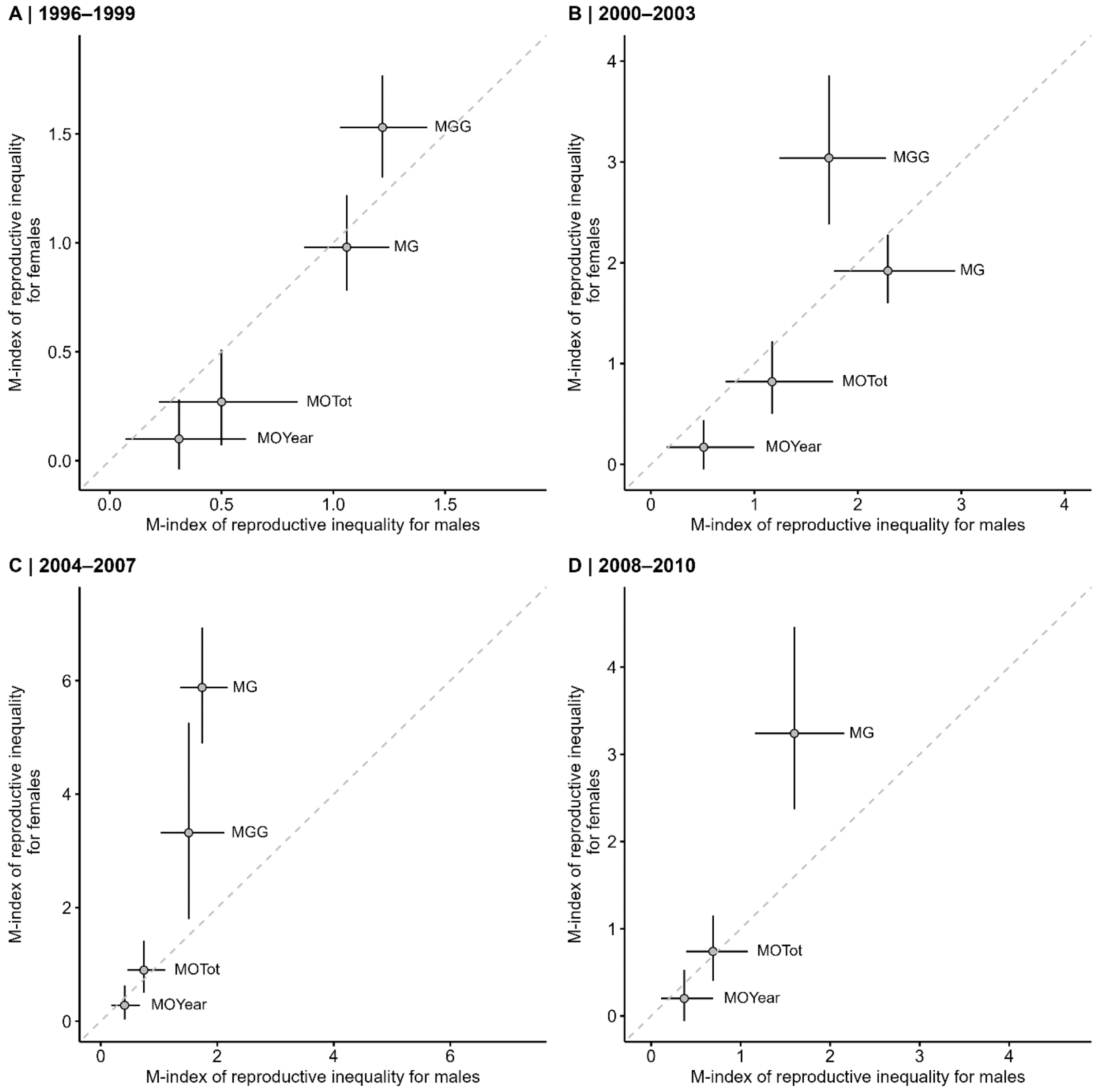

Fig. S4. Cohort-specific comparison of reproductive inequality in female and male spotted hyenas. Relationship between male (x-axis) and female (y-axis) M-indices of reproductive inequality for four measures of reproductive success: annual number of offspring (M_{OYear}), lifetime number of offspring (M_{OTot}), grandoffspring (M_G), and great-grandoffspring (M_{GG}), calculated separately for four cohorts: (**A**) 1996–1999, (**B**) 2000–2003, (**C**) 2004–2007, and (**D**) 2008–2010. Within each cohort, only individuals who initiated their reproductive career in the same time window are compared, ensuring demographic and ecological contemporaneity. For great-grandoffspring (M_{GG}), analyses used reduced sample sizes in panel **C** and are absent from panel **D**, as the 2008–2010 cohort falls outside the completeness threshold (see Methods). Filled circles are point estimates; horizontal and vertical lines indicate 95% credible intervals. The dashed diagonal line represents equal M-indices in the two sexes**.**

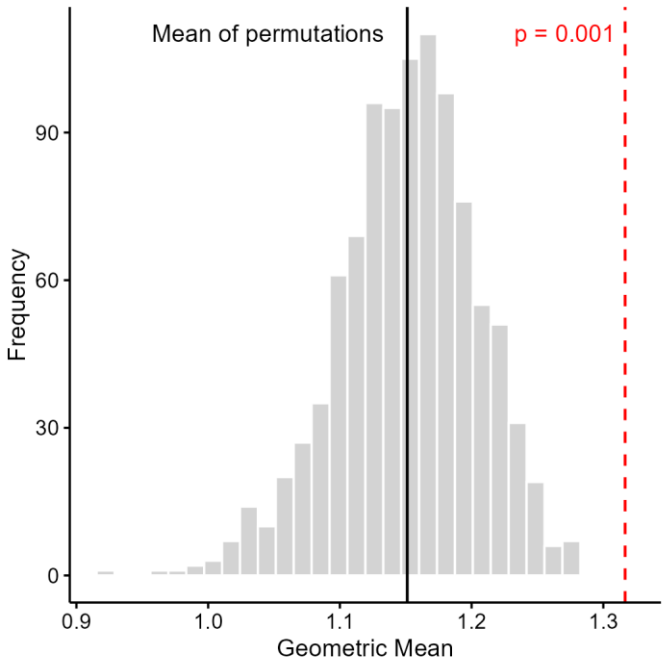

Fig. S5. Permutation distribution of geometric means for maternal-line rank transmission. The histogram shows the distribution of geometric means across permutations of maternal identity within clans and two-year retrospective birth cohorts, under a null model excluding social rank inheritance. The solid black line indicates the mean of the permutation distribution, and the dashed red line marks the observed geometric mean from the actual maternal lineages. The observed value lies near the upper tail of the null distribution, suggesting that the intergenerational continuity in rank is stronger than expected by chance. The corresponding permutation p-value is displayed on the plot.

Table S1. Parameter estimates from the zero-inflated negative binomial (ZINB) model of grandoffspring count.

Component	Term	Estimate	SE	chisq	df	p-value
count	Intercept	-0.099	0.197			
count	Sex (male)	0.877	0.266	2.687	1	0.101
count	Log number of offspring	1.472	0.107	258.382	1	< 0.001
count	Completeness	0.072	0.073	0.290	1	0.590
count	Sex (male) × Log number of offspring	-0.424	0.144	8.623	1	0.003
count	Sex (male) × Completeness	-0.079	0.095	0.696	1	0.404
zi	Intercept	-24.191	3,966.872			
zi	Completeness	46.893	8,168.936			

Results are shown separately for the conditional (count) and zero-inflation (zi) components. Estimates are presented on the model link scale (log for the count component, logit for the zero-inflation component). Likelihood-ratio test (χ^2) statistics and associated p-values are reported in place of z-tests. Completeness: the proportion of an individual's offspring deceased by the end of the study period, included as a covariate to account for variation in the completeness of grandoffspring counts across individuals (see Supplementary Text, p2)

Table S2. Descriptive statistics of expected genetic contribution for all individuals and separated by sex

Sex	N	Mean	Median	SD	Min	Max	Q1	Q3
Female	239	1.42	0.26	2.79	0	24.11	0	1.67
Male	167	1.94	1.00	2.52	0	12.88	0	2.69
All	406	1.63	0.61	2.69	0	24.11	0	2.10

REFERENCES

1. A. J. Bateman, Intra-sexual selection in *Drosophila*. *Heredity* **2**, 349–368 (1948).

2. C. Darwin, *The Descent of Man, and Selection in Relation to Sex* (John Murray, London, 1871).

3. T. Janicke, Anisogamy and the Darwin-Bateman paradigm. *Evol. Lett.* **8**, 756–760 (2024).

4. D. A. Dewsbury, The Darwin-Bateman paradigm in historical context. *Integr. Comp. Biol.* **45**, 831–837 (2005).

5. T. Janicke, I. K. Häderer, M. J. Lajeunesse, N. Anthes, Darwinian sex roles confirmed across the animal kingdom. *Sci. Adv.* **2**, e1500983 (2016).

6. T. H. Clutton-Brock, *Reproductive Success: Studies of Individual Variation in Contrasting Breeding Systems* (University of Chicago Press, 1988).

7. C. T. Ross, P. L. Hooper, J. E. Smith, A. V. Jaeggi, E. A. Smith, S. Gavrilets, F. T. Zohora, J. Ziker, D. Xygalatas, E. E. Wroblewski, B. Wood, B. Winterhalder, K. P. Willführ, A. K. Willard, K. Walker, C. Von Rueden, E. Voland, C. Valeggia, B. Vaitla, S. Urlacher, M. Towner, C.-Y. Sum, L. S. Sugiyama, K. B. Strier, K. Starkweather, D. Major-Smith, M. Shenk, R. Sear, E. Seabright, R. Schacht, B. Scelza, S. Scaggs, J. Salerno, C. Revilla-Minaya, D. Redhead, A. Pusey, B. G. Purzycki, E. A. Power, A. Pisor, J. Pettay, S. Perry, A. E. Page, L. Pacheco-Cobos, K. Oths, S.-Y. Oh, D. Nolin, D. Nettle, C. Moya, A. B. Migliano, K. J. Mertens, R. A. McNamara, R. McElreath, S. Mattison, E. Massengill, F. Marlowe, F. Madimenos, S. Macfarlan, V. Lummaa, R. Lizarralde, R. Liu, M. A. Liebert, S. Lew-Levy, P. Leslie, J. Lanning, K. Kramer, J. Koster, H. S. Kaplan, B. Jamsranjav, A. M. Hurtado, K. Hill, B. Hewlett, S. Helle, T. Headland, J. Headland, M. Gurven, G. Grimalda, R. Greaves, C. D. Golden, I. Godoy, M. Gibson, C. E. Mouden, M. Dyble, P. Draper, S. Downey, A. L. DeMarco, H. E. Davis, S. Crabtree, C. Cortez, H. Colleran, E. Cohen, G. Clark, J. Clark, M. A. Caudell, C. E. Carminito, J. Bunce, A. Boyette, S. Bowles, T. Blumenfield, B. Beheim, S. Beckerman, Q. Atkinson, C. Apicella, N. Alam, M. B. Mulder, Reproductive inequality in humans and other mammals. *Proc. Natl. Acad. Sci. U.S.A.* **120**, e2220124120 (2023).

8. K. Fritzsche, J. M. Henshaw, B. D. Johnson, A. G. Jones, The 150th anniversary of The Descent of Man: Darwin and the impact of sex-role reversal on sexual selection research. *Biol. J. Linn. Soc.* **134**, 525–540 (2021).

9. A. G. Jones, J. C. Avise, Mating systems and sexual selection in male-pregnant pipefishes and seahorses: Insights from microsatellite-based studies of maternity. *J. Hered.* **92**, 150–158 (2001).

10. G. Lopez-Nava, I. Safari, C. Küpper, W. Goymann, Sex-role reversal and the Bateman gradient in coucals—Females benefit from mating with multiple partners. *Proc. Biol. Sci.* **293**, 20252860 (2026).

11. T. H. Clutton-Brock, S. J. Hodge, G. Spong, A. F. Russell, N. R. Jordan, N. C. Bennett, L. L. Sharpe, M. B. Manser, Intrasexual competition and sexual selection in cooperative mammals. *Nature* **444**, 1065–1068 (2006).

12. D. R. Rubenstein, I. J. Lovette, Reproductive skew and selection on female ornamentation in social species. *Nature* **462**, 786–789 (2009).

13. J. E. Smith, B. Natterson-Horowitz, M. E. Alfaro, The nature of privilege: Intergenerational wealth in animal societies. *Behav. Ecol.* **33**, 1–6 (2022).

14. E. D. Strauss, D. Shizuka, The ecology of wealth inequality in animal societies. *Proc. Biol. Sci.* **289**, 20220500 (2022).

15. M. Borgerhoff Mulder, S. Bowles, T. Hertz, A. Bell, J. Beise, G. Clark, I. Fazzio, M. Gurven, K. Hill, P. L. Hooper, W. Irons, H. Kaplan, D. Leonetti, B. Low, F. Marlowe, R. McElreath, S. Naidu, D. Nolin, P. Piraino, R. Quinlan, E. Schniter, R. Sear, M. Shenk, E. A. Smith, C. von Rueden, P. Wiessner, Intergenerational wealth transmission and the dynamics of inequality in small-scale societies. *Science* **326**, 682–688 (2009).

16. M. L. East, O. P. Höner, B. Wachter, K. Wilhelm, T. Burke, H. Hofer, Maternal effects on offspring social status in spotted hyenas. *Behav. Ecol.* **20**, 478–483 (2009).

17. L. Ellis, Dominance and reproductive success among nonhuman animals: A cross-species comparison. *Ethol. Sociobiol.* **16**, 257–333 (1995).

18. A. Ilany, K. E. Holekamp, E. Akçay, Rank-dependent social inheritance determines social network structure in spotted hyenas. *Science* **373**, 348–352 (2021).

19. Shivani, E. Huchard, D. Lukas, The effect of dominance rank on female reproductive success in social mammals. *Peer Community J.* **2**, e48 (2022).

20. C. R. von Rueden, A. V. Jaeggi, Men's status and reproductive success in 33 nonindustrial societies: Effects of subsistence, marriage system, and reproductive strategy. *Proc. Natl. Acad. Sci. U.S.A.* **113**, 10824–10829 (2016).

21. K. Price, S. Boutin, Territorial bequeathal by red squirrel mothers. *Behav. Ecol.* **4**, 144–150 (1993).

22. J. E. Ragsdale, Reproductive skew theory extended: The effect of resource inheritance on social organization. *Evol. Ecol. Res.* **1**, 859–874 (1999).

23. G. E. Woolfenden, J. W. Fitzpatrick, The inheritance of territory in group-breeding birds. *Bioscience* **28**, 104–108 (1978).

24. D. J. Emlen, The evolution of animal weapons. *Annu. Rev. Ecol. Evol. Syst.* **39**, 387–413 (2008).

25. T. A. Mousseau, D. A. Roff, Natural selection and the heritability of fitness components. *Heredity* **59**, 181–197 (1987).

26. C. Reuland, L. W. Simmons, S. Lüpold, J. L. Fitzpatrick, Weapons evolve faster than sperm in bovids and cervids. *Cells* **10**, 1062 (2021).

27. M. Borgerhoff Mulder, M. C. Towner, R. Baldini, B. A. Beheim, S. Bowles, H. Colleran, M. Gurven, K. L. Kramer, S. M. Mattison, D. A. Nolin, B. A. Scelza, E. Schniter, R. Sear, M. K.

Shenk, E. Voland, J. Ziker, Differences between sons and daughters in the intergenerational transmission of wealth. *Philos. Trans. R. Soc.Lond. B Biol. Sci.* **374**, 20180076 (2019).

28. K. E. Holekamp, L. Smale, Dominance acquisition during mammalian social development: The "inheritance" of maternal rank. *Am. Zool.* **31**, 306–317 (1991).

29. S. Kawamura, The matriarchal social order in the Minoo-B Group. *Primates* **1**, 149–156 (1958).

30. S. Shennan, Property and wealth inequality as cultural niche construction. *Philos. Trans. R. Soc. Lond. B Biol. Sci.* **366**, 918–926 (2011).

31. D. Lukas, T. Clutton-Brock, Costs of mating competition limit male lifetime breeding success in polygynous mammals. *Proc. Biol. Sci.* **281**, 20140418 (2014).

32. H. Hofer, M. L. East, Behavioral processes and costs of co-existence in female spotted hyenas: A life history perspective. *Evol. Ecol.* **17**, 315–331 (2003).

33. K. E. Holekamp, L. Smale, Ontogeny of dominance in free-living spotted hyaenas: Juvenile rank relations with other immature individuals. *Anim. Behav.* **46**, 451–466 (1993).

34. K. E. Holekamp, L. Smale, M. Szykman, Rank and reproduction in the female spotted hyaena. *Reproduction* **108**, 229–237 (1996).

35. E. D. Strauss, D. Shizuka, K. E. Holekamp, Juvenile rank acquisition is associated with fitness independent of adult rank. *Proc. Biol. Sci.* **287**, 20192969 (2020).

36. M. East, H. Hofer, A. Turk, Functions of birth dens in spotted hyaenas (*Crocuta crocuta*). *J. Zool.* **219**, 690–697 (1989).

37. M. Gicquel, M. L. East, H. Hofer, S. Benhaiem, Early-life adversity predicts performance and fitness in a wild social carnivore. *J. Anim. Ecol.* **91**, 2074–2086 (2022).

38. K. E. Holekamp, E. D. Strauss, Reproduction within a hierarchical society from a female's perspective. *Integr. Comp. Biol.* **60**, 753–764 (2020).

39. O. P. Höner, B. Wachter, H. Hofer, K. Wilhelm, D. Thierer, F. Trillmich, T. Burke, M. L. East, The fitness of dispersing spotted hyaena sons is influenced by maternal social status. *Nat. Commun.* **1**, 60 (2010).

40. O. P. Höner, B. Wachter, M. L. East, W. J. Streich, K. Wilhelm, T. Burke, H. Hofer, Female mate-choice drives the evolution of male-biased dispersal in a social mammal. *Nature* **448**, 798–801 (2007).

41. E. Davidian, A. Courtiol, B. Wachter, H. Hofer, O. P. Höner, Why do some males choose to breed at home when most other males disperse? *Sci. Adv.* **2**, e1501236 (2016).

42. C. T. Ross, A. V. Jaeggi, M. Borgerhoff Mulder, J. E. Smith, E. A. Smith, S. Gavrilets, P. L. Hooper, The multinomial index: A robust measure of reproductive skew. *Proc. Biol. Sci.* **287**, 20202025 (2020).

43. M. O. Lorenz, Methods of measuring the concentration of wealth. *Publ. Am. Stat. Assoc.* **9**, 209–219 (1905).

44. T. Bonnet, M. B. Morrissey, P. de Villemereuil, S. C. Alberts, P. Arcese, L. D. Bailey, S. Boutin, P. Brekke, L. J. N. Brent, G. Camenisch, A. Charmantier, T. H. Clutton-Brock, A. Cockburn, D. W. Coltman, A. Courtiol, E. Davidian, S. R. Evans, J. G. Ewen, M. Festa-Bianchet, C. de Franceschi, L. Gustafsson, O. P. Höner, T. M. Houslay, L. F. Keller, M. Manser, A. G. McAdam, E. McLean, P. Nietlisbach, H. L. Osmond, J. M. Pemberton, E. Postma, J. M. Reid, A. Rutschmann, A. W. Santure, B. C. Sheldon, J. Slate, C. Teplitsky, M. E. Visser, B. Wachter, L. E. B. Kruuk, Genetic variance in fitness indicates rapid contemporary adaptive evolution in wild animals. *Science* **376**, 1012–1016 (2022).

45. G. Kosova, M. Abney, C. Ober, Heritability of reproductive fitness traits in a human population. *Proc. Natl. Acad. Sci. U.S.A.* **107**, 1772–1778 (2010).

46. J. Merilä, B. C. Sheldon, Lifetime reproductive success and heritability in nature. *Am. Nat.* **155**, 301–310 (2000).

47. K. E. Holekamp, J. E. Smith, C. C. Strelioff, R. C. Van Horn, H. E. Watts, Society, demography and genetic structure in the spotted hyena. *Mol. Ecol.* **21**, 613–632 (2012).

48. E. Heyer, R. Chaix, S. Pavard, F. Austerlitz, Sex-specific demographic behaviours that shape human genomic variation. *Mol. Ecol.* **21**, 597–612 (2012).

49. O. P. Höner, B. Wachter, M. L. East, V. A. Runyoro, H. Hofer, The effect of prey abundance and foraging tactics on the population dynamics of a social, territorial carnivore, the spotted hyena. *Oikos* **108**, 544–554 (2005).

50. C. Vullioud, E. Davidian, B. Wachter, F. Rousset, A. Courtiol, O. P. Höner, Social support drives female dominance in the spotted hyaena. *Nat. Ecol. Evol.* **3**, 71–76 (2019).

51. M. E. Hauber, E. A. Lacey, Bateman's principle in cooperatively breeding vertebrates: The effects of non-breeding alloparents on variability in female and male reproductive success. *Integr. Comp. Biol.* **45**, 903–914 (2005).

52. D. R. Rubenstein, Temporal but not spatial environmental variation drives adaptive offspring sex allocation in a plural cooperative breeder. *Am. Nat.* **170**, 155–165 (2007).

53. T. Clutton-Brock, Sexual selection in males and females. *Science* **318**, 1882–1885 (2007).

54. E. D. Strauss, K. E. Holekamp, Social alliances improve rank and fitness in convention-based societies. *Proc. Natl. Acad. Sci. U.S.A.* **116**, 8919–8924 (2019).

55. D. Lukas, E. Huchard, The evolution of infanticide by females in mammals. *Philos. Trans. R. Soc. Lond. B Biol. Sci.* **374**, 20180075 (2019).

56. A. K. Brown, M. O. Pioon, K. E. Holekamp, E. D. Strauss, Infanticide by females is a leading source of juvenile mortality in a large social carnivore. *Am. Nat.* **198**, 642–652 (2021).

57. R. M. Hare, L. W. Simmons, Sexual selection and its evolutionary consequences in female animals. *Biol. Rev. Camb. Philos. Soc.* **94**, 929–956 (2019).

58. T. H. Clutton-Brock, E. Huchard, Social competition and selection in males and females. *Philos. Trans. R. Soc. B Biol. Sci.* **368**, 20130074 (2013).

59. P. Stockley, J. Bro-Jørgensen, Female competition and its evolutionary consequences in mammals. *Biol. Rev.* **86**, 341–366 (2011).

60. K. Karlsson Green, J. A. Madjidian, Active males, reactive females: Stereotypic sex roles in sexual conflict research? *Anim. Behav.* **81**, 901–907 (2011).

61. E. Davidian, B. Wachter, I. Heckmann, M. Dehnhard, H. Hofer, O. P. Höner, The interplay between social rank, physiological constraints and investment in courtship in male spotted hyenas. *Funct. Ecol.* **35**, 635–649 (2021).

62. E. Heyer, A. Sibert, F. Austerlitz, Cultural transmission of fitness: Genes take the fast lane. *Trends Genet.* **21**, 234–239 (2005).

63. N. Chen, I. Juric, E. J. Cosgrove, R. Bowman, J. W. Fitzpatrick, S. J. Schoech, A. G. Clark, G. Coop, Allele frequency dynamics in a pedigreed natural population. *Proc. Natl. Acad. Sci. U.S.A.* **116**, 2158–2164 (2019).

64. M. J. Kelly, Lineage loss in Serengeti cheetahs: Consequences of high reproductive variance and heritability of fitness on effective population size. *Conserv. Biol.* **15**, 137–147 (2001).

65. J. F. Crow, Breeding structure of populations. II. Effective population number, in *Statistics and Mathematics in Biology* (Iowa State College Press, Ames, Iowa, 1954), pp. 543–556.

66. R. S. Waples, The N_e/N ratio in applied conservation. *Evol. Appl.* **17**, e13695 (2024).

67. S. Wright, Evolution in mendelian populations. *Genetics* **16**, 97–159 (1931).

68. B. Charlesworth, Effective population size and patterns of molecular evolution and variation. *Nat. Rev. Genet.* **10**, 195–205 (2009).

69. L. Smale, S. Nunes, K. E. Holekamp, "Sexually Dimorphic Dispersal in Mammals: Patterns, Causes, and Consequences," in *Advances in the Study of Behavior*, P. J. B. Slater, J. S.

Rosenblatt, C. T. Snowdon, M. Milinski, Eds. (Academic Press, San Diego, 1997), vol. **26**, pp. 181–250.

70. M. L. East, T. Burke, K. Wilhelm, C. Greig, H. Hofer, Sexual conflicts in spotted hyenas: Male and female mating tactics and their reproductive outcome with respect to age, social status and tenure. *Proc. Biol. Sci.* **270**, 1247–1254 (2003).

71. R. Mace, Biased parental investment and reproductive success in Gabbra pastoralists. *Behav. Ecol. Sociobiol.* **38**, 75–81 (1996).

72. N. Kutsukake, Matrilineal rank inheritance varies with absolute rank in Japanese macaques. *Primates* **41**, 321–335 (2000).

73. M. A. van Noordwijk, C. P. van Schaik, The effects of dominance rank and group size on female lifetime reproductive success in wild long-tailed macaques, *Macaca fascicularis*. *Primates* **40**, 105–130 (1999).

74. S. K. Wasser, G. W. Norton, S. Kleindorfer, R. J. Rhine, Population trend alters the effects of maternal dominance rank on lifetime reproductive success in yellow baboons (*Papio cynocephalus*). *Behav. Ecol. Sociobiol.* **56**, 338–345 (2004).

75. R. A. Blersch, T. R. Bonnell, M. Clarke, M. J. Dostie, M. Lucas, J. Jarrett, R. McFarland, C. Nord, A. Takahashi, S. Varsanyi, C. Vilette, C. Young, L. Barrett, S. P. Henzi, Maternal social position and survival to weaning in arid-country vervet monkeys. *Am. J. Biol. Anthropol.* **181**, 3–9 (2023).

76. S. B. Hrdy, D. S. Judge, Darwin and the puzzle of primogeniture. *Hum. Nat.* **4**, 1–45 (1993).

77. E. M. Swanson, T. L. McElhinny, I. Dworkin, M. L. Weldele, S. E. Glickman, K. E. Holekamp, Ontogeny of sexual size dimorphism in the spotted hyena (*Crocuta crocuta*). *J. Mammal.* **94**, 1298–1310 (2013).

78. É. Danchin, A. Charmantier, F. A. Champagne, A. Mesoudi, B. Pujol, S. Blanchet, Beyond DNA: Integrating inclusive inheritance into an extended theory of evolution. *Nat. Rev. Genet.* **12**, 475–486 (2011).

79. S. Benhaiem, H. Hofer, S. Kramer-Schadt, E. Brunner, M. L. East, Sibling rivalry: Training effects, emergence of dominance and incomplete control. *Proc. Biol. Sci.* **279**, 3727–3735 (2012).

80. H. Hofer, M. L. East, Siblicide in Serengeti spotted hyenas: A long-term study of maternal input and cub survival. *Behav. Ecol. Sociobiol.* **62**, 341–351 (2008).

81. M. L. East, Male spotted hyenas (*Crocuta crocuta*) queue for status in social groups dominated by females. *Behav. Ecol.* **12**, 558–568 (2001).

82. L. Smale, L. G. Frank, K. E. Holekamp, Ontogeny of dominance in free-living spotted hyaenas: Juvenile rank relations with adult females and immigrant males. *Anim. Behav.* **46**, 467–477 (1993).

83. K. Wilhelm, D. A. Dawson, L. K. Gentle, G. F. Horsfield, C. Schlötterer, C. Greig, M. East, H. Hofer, D. Tautz, T. Burke, Characterization of spotted hyena, *Crocuta crocuta* microsatellite loci. *Mol. Ecol. Notes* **3**, 360–362 (2003).

84. S. T. Kalinowski, M. L. Taper, T. C. Marshall, Revising how the computer program CERVUS accommodates genotyping error increases success in paternity assignment. *Mol. Ecol.* **16**, 1099–1106 (2007).

85. L. Bailey, C. Vullioud, E. Donati, Z. Li, I. Heckmann, A. Courtiol, hyenaR: Functions for analyzing hyena data from Ngorongoro Crater, version 0.10.0.9000 (2025); https://github.com/hyenaproject/drat.

86. M. E. Brooks, K. Kristensen, K. J. van Benthem, A. Magnusson, C. W. Berg, A. Nielsen, H. J. Skaug, M. Mächler, B. M. Bolker, glmmTMB balances speed and flexibility among packages for zero-inflated generalized linear mixed modeling. *R J.* **9**, 378–400 (2017).

87. F. Hartig, DHARMa: Residual diagnostics for hierarchical (multi-Level/mixed) regression models (2024); https://CRAN.R-project.org/package=DHARMa.

88. C. Gini, Measurement of inequality of incomes. *Econ. J.* **31**, 124–126 (1921).

89. A. Signorell, DescTools: Tools for descriptive statistics (2025); https://CRAN.R-project.org/package=DescTools.

90. J. Fox, S. Weisberg, *An R Companion to Applied Regression* (Sage, Thousand Oaks CA, ed. 3, 2019); https://john-fox.ca/Companion/.

91. C. Blötner, diffcor: Fisher's *z*-tests concerning differences between correlations (2024); https://CRAN.R-project.org/package=diffcor.

92. B. Phipson, G. K. Smyth, Permutation p-values should never be zero: Calculating exact p-values when permutations are randomly drawn. *Stat. Appl. Genet. Mol. Biol.* **9**, Article 39 (2010).

93. S. Xu, M. Chen, T. Feng, L. Zhan, L. Zhou, G. Yu, Use ggbreak to effectively utilize plotting space to deal with large datasets and outliers. *Front. Genet.* **12**, 774846 (2021).
